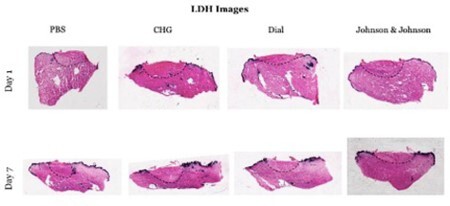# 811 The Cytotoxic Effect of CHG, Dial, and Johnson & Johnson on Human Burn Wounds

**DOI:** 10.1093/jbcr/irae036.351

**Published:** 2024-04-17

**Authors:** Rabia Ahmed, Jocelyn C Zajac, Aiping Liu, Joana Pashaj, Angela Gibson

**Affiliations:** University of Wisconsin - Madison, Madison, Wisconsin; University of Wisconsin School of Medicine and Public Health, Madison, WI; University of Wisconsin - Madison, Madison, Wisconsin; University of Wisconsin School of Medicine and Public Health, Madison, WI; University of Wisconsin - Madison, Madison, Wisconsin; University of Wisconsin School of Medicine and Public Health, Madison, WI; University of Wisconsin - Madison, Madison, Wisconsin; University of Wisconsin School of Medicine and Public Health, Madison, WI; University of Wisconsin - Madison, Madison, Wisconsin; University of Wisconsin School of Medicine and Public Health, Madison, WI

## Abstract

**Introduction:**

Chlorhexidine gluconate (CHG) and soaps are commonly used to prevent burn wound infections in patients. However, it is thought that these antiseptic reagents may be cytotoxic to human skin and may impede proper healing of burn wounds. We have previously shown profound cytotoxicity of CHG in excisional wounds using an ex vivo human skin model. The purpose of this study was to observe the effects of CHG, Dial, and Johnson & Johnson (J&J) baby soap on cytotoxicity using an ex vivo human skin model of burn.

**Methods:**

Partial thickness burns were created on the skin samples using a customized burn device at 150 °C for 6 seconds (n =3 per condition per time point). The burn wounds were treated daily with either CHG, Dial, J&J, or PBS (phosphate buffered saline) for 20 seconds using a cotton tipped applicator and then aspirated and rinsed using 1 milliliter of PBS 3 times to mimic clinical practice. The tissue samples were then cultured for 7 days in 37 °C with 5% CO2. We used PBS as positive control for cell viability and full thickness boiled samples as negative control for 100% of cell death. The cytotoxicity of CHG, Dial, J&J, was measured using lactate dehydrogenase (LDH) staining and an MTT viability assay on days 1 and 7. A two-way ANOVA statistical test was performed on the MTT with additional Tukey analysis between time points and treatment groups.

**Results:**

In this ex vivo human skin model, we observed progressive loss of viability of PBS treated burn wounds indicative of burn progression. For better comparison among groups, in the MTT assays, we corrected for weight and normalized to PBS. We found a significant difference in viability between CHG and PBS on day 7 (p=.0388). In the samples stained with LDH: CHG, Dial, J&J, and PBS all show visible viable tissue on day 1. On day 7, while all samples have viable cells outside of the area of burn injury, the viability has decreased visibly in the cells surrounding the original burn region on both CHG and Dial treated samples. However, there were no significant differences in the overall amount of viable tissue between days or treatment groups.

**Conclusions:**

This study shows that CHG is toxic to the human skin starting from day 1 and leads to loss of viable skin cells extending outside of the original burn region by day 7. When applied to burn wounds for up to 7 days, J&J is less cytotoxic to the skin cells compared to the Dial soap. Additionally, this model may be utilized to study burn wound progression in isolation of perfusion to identify non-vascular mechanisms of burn wound progression.

**Applicability of Research to Practice:**

Although CHG is currently standard-of-care for many burn centers, physicians should take the cytotoxicity of CHG into consideration when using CHG to prevent burn wound infection. Compared to Dial soap, J&J soap may be a good alternative to CHG, because J&J is less cytotoxic to normal skin cells.